# The IGF Signalling Axis in Lung Cancer: Clinical Significance and Therapeutic Challenges

**DOI:** 10.1111/jcmm.70540

**Published:** 2025-04-05

**Authors:** Kostas A. Papavassiliou, Amalia A. Sofianidi, Kyriaki Cholidou, Athanasios G. Papavassiliou

**Affiliations:** ^1^ First University Department of Respiratory Medicine ‘Sotiria’ Chest Hospital, Medical School, National and Kapodistrian University of Athens Athens Greece; ^2^ Department of Biological Chemistry Medical School, National and Kapodistrian University of Athens Athens Greece

**Keywords:** IGF signalling axis, lung cancer, targeted therapy

Lung cancer ranks as the leading cause of cancer‐related mortality worldwide, with an estimated 1.8 million deaths in 2022 [1]. The medical community has not managed to tame this deadly malignancy so far, which is mainly attributed to its high aggressiveness and our deficient understanding of its distinct biological features. Recently, metabolic reprogramming has emerged as an important cancer hallmark [2], helping us better understand the profile of the tumour. In this vein, the insulin‐like growth factor (IGF) axis, comprising IGF‐1/IGF‐2, related receptors (IGF‐1R/‐2R), and high‐affinity binding proteins (IGFBP 1‐6), has become apparent as a central player in lung cancer growth, invasion and metastasis [3]. Herein, we provide an update on the clinical significance of the IGF signalling axis in lung cancer, highlighting the latest advancements that have developed in the past few years.

IGFs are polypeptides that serve as growth factors, tethering to IGF receptors and initiating signalling cascades. The IGF axis consists of two main ligands, IGF‐1 and IGF‐2, the associated cellular receptors IGF‐1R and IGF‐2R, and their binding soluble plasma proteins (IGFBPs). The interaction of the ligands with their receptors leads to the activation of the phosphoinositide‐3‐kinase (PI3K)–protein kinase B (PKB)/AKT and mitogen‐activated protein kinase (MAPK) signalling pathways. On the other hand, IGFBPs bind IGFs and hinder them from tethering to their receptors and activating downstream signalling cascades [4]. Structurally similar to insulin, IGFs control the development, differentiation, and proliferation of normal cells across the lifespan [4]. Numerous studies have lately revealed that the IGF axis is intimately implicated in lung carcinogenesis [3]. This observation is not a new one; the hypothesis that the IGF axis is involved in the tumorigenesis of several malignancies roots more than 20 years ago (e.g., [5]).

It is nowadays well established that the IGF signalling cascade is upregulated in lung cancer [3, 6, 7]. Studies have demonstrated that IGF‐1R boosts the metastatic potential of lung cancer cells by enabling epithelial‐to‐mesenchymal transition (EMT) and promoting DNA damage processes [8]. A novel study also showed that a tobacco smoke‐specific carcinogen, namely nitrosamine 4‐(methylnitrosamino)‐1‐(3‐pyridyl)‐1‐butanone (nicotine‐derived nitrosamine ketone, NNK), activates IGF signalling, inducing lung tumorigenesis. NNK fosters Src‐mediated signal transducer and activator of transcription 3 (STAT3) potentiation and increased release of calcium through the angiotensin II (AngII) receptor type 1 (AGTR1, AT1 receptor)−phospholipase C (PLC) axis, leading to transcriptional upregulation of IGF‐2 and lung tumorigenesis in tobacco smokers [9]. Remarkably, it was also found that IGF‐1R acts in the lung tumour microenvironment (TME) sustaining inflammation and tumour‐associated immunosuppression [8]. Targeting the IGF axis by suppressing IGF‐1 levels could boost tumour‐specific immunity and open new avenues for the exploitation of the programmed death‐ligand 1 (PD‐L1)−programmed death protein 1 (PD‐1) axis in lung cancer therapeutics [10]. The clinical importance of the IGF axis in lung cancer is further enhanced by a recent study that revealed a connection between diabetes and lung cancer through this axis [11]. Patients with diabetes have increased levels of insulin, which stimulates liver cells to produce IGF‐1. IGF‐1, in turn, controls the proliferation, differentiation and apoptosis of lung cancer cells by triggering several metabolic and mitotic signalling cascades [11].

The role of the IGF signalling pathway is decisive in developing tumour chemoresistance in lung cancer [12]. Intriguingly, the IGF axis is implicated in developing resistance to osimertinib, a third‐generation tyrosine kinase inhibitor (TKI), in epidermal growth factor receptor (EGFR)‐mutant lung cancer [13, 14]. Up until now, it was accepted that the loss of IGFBP3 contributes to third‐generation TKI resistance by upregulating IGF‐1R activation [13]. However, a new study introduced the IGF‐2 autocrine‐mediated IGF‐1R potentiation as a nongenetic mechanism of osimertinib resistance in lung cancer [14]. What is novel in the field is the entanglement of cancer‐associated fibroblasts (CAFs) in IGF signalling and the subsequent drug sensitisation of lung cancer cells [15]. Compared to normal fibroblasts, CAFs secrete lower amounts of IGFs, but higher amounts of IGFBPs, which hamper IGF signalling. Furthermore, IGFBPs engage with integrins and decrease focal adhesion kinase (FAK) signalling, which is essential for the survival of lung tumour cells in response to EGFR‐targeted therapy. Correspondingly, the response of lung cancer cells to osimertinib is ameliorated [15]. In this context, another research introduced circular RNAs (circRNAs) as pivotal regulators of osimertinib resistance in non‐small cell lung cancer (NSCLC). It was demonstrated that the expression of circ_PPAPDC1A is upregulated in NSCLC, exerting a significant oncogenic role by interacting with miR‐30a‐3p, augmenting the expression of IGF‐1R, and activating the IGF‐1R–PI3K–AKT–mammalian target of rapamycin (mTOR) pathway [16].

The implication of the IGF‐1R signalling axis in lung cancer progression and resistance to conventional therapy has defined this axis as a promising target in lung cancer therapeutics. Apart from monoclonal antibodies (mAbs) and TKIs targeting IGF‐1R, antibody‐drug conjugates (ADCs) are currently being examined. W0101, an IGF‐1R ADC, inhibited tumour growth in lung cancer cell lines expressing high levels of IGF‐1R [17]. Of note, Src and AXL (a member of the TAM family of receptor tyrosine kinases (RTKs)) kinases have been found to bestow resistance on anti‐IGF‐1R therapies [18, 19]. Researchers tried to create a compound that simultaneously blocks IGF‐1R, Src, and AXL and developed LL6 (a phenylpyrazolo[3,4‐*d*]pyrimidine‐based, small‐molecule kinase inhibitor) that suppressed lung tumour growth both in vitro and in vivo [20]. When exploring natural compounds, the Chinese herb breviscapine (BVP; a mixture of flavonoid glycosides) was found to inhibit NSCLC growth by stimulating apoptosis via reactive oxygen species (ROS)‐mediated upregulation of IGFBP4 [21]. Combinatorial treatments have also been preclinically explored. BMS‐754807, a small‐molecule inhibitor targeting the IGF‐1R and insulin receptor (IR), in combination with the TKI dasatinib (a multi‐targeted TKI) impedes lung cancer cell proliferation and tumour growth in vitro [22]. In the clinical setting, a phase I trial assessed the combination of xentuzumab, an IGF‐1 and IGF‐2 neutralising mAb, and the EGFR TKI afatinib in patients with previously treated EGFR‐mutant NSCLC. However, the combination did not reveal increased therapeutic efficacy after progression on afatinib [23].

Nevertheless, challenges exist that cannot be ignored. IGF‐1/2 bind to IGF‐1R, initiating signalling cascades. In contrast, insulin binds to the IR, resulting in controlled glucose metabolism. IGF‐1R dimers and IR dimers can merge, forming hybrid receptors that bind both molecules and trigger metabolic and mitogenic effects. Considering that the targeted inhibition of IGF‐1R is associated with major adverse events, as it can cause metabolic aberrations like hyperglycemia and insulin deregulation [24]. Due to the complexity of the receptors themselves, downstream molecules in the IGF cascade could also be explored, which, if targeted, may enrich our therapeutic arsenal against lung cancer.

Additionally, careful patient selection is another challenge that needs to be addressed in clinical trials evaluating IGF‐targeted therapy. Several clinical trials have failed to establish IGF‐targeted therapies as superior therapeutic modalities compared to conventional regimens. For instance, as mentioned before, when adding xentuzumab to afatinib in patients with NSCLC, there is no significant benefit in terms of objective response [23]. Notably, as many successful targeted anti‐cancer therapies rely upon the use of predictive biomarkers, delving deeper into this area of investigation is crucial for determining the most effectual application of IGF/IGF‐1R inhibitors as a lung cancer therapy [25]. As proof, increased plasma levels of IGF‐1 or increased IGF‐1R expression in NSCLC tumours were linked to resistance to anti‐PD‐1 immunotherapy [10]. Carefully selecting patients with high serum levels of IGF‐1 or high IGF‐1R expression for recruitment in clinical trials evaluating immune checkpoint inhibitors (ICIs) combined with IGF axis blockade could yield more favourable results.

In summary, even though we have unearthed the therapeutic potential of targeting the IGF axis, we have not yet managed to utilise this discovery effectively in clinical practice. Continued exploration of the intricate interactions between the IGF signalling axis–cognate druggable transcriptional effectors [26] and the TME could unveil new therapeutic opportunities, especially if we could decipher the exact role of CAFs and other TME components in this vital lung cancer signalling “circuitry”.

## Author Contributions

**Kostas A. Papavassiliou:** conceptualization (lead), data curation (lead), writing – original draft (lead). **Amalia A. Sofianidi:** conceptualization (equal), data curation (equal), writing – original draft (equal). **Kyriaki Cholidou:** conceptualization (equal), data curation (equal), writing – original draft (equal). **Athanasios G. Papavassiliou:** conceptualization (lead), data curation (lead), supervision (lead), writing – review and editing (lead).

## Conflicts of Interest

The authors declare no conflicts of interest.

## Data Availability

The authors have nothing to report.
